# Involvement of DNA Damage Response Pathways in Hepatocellular Carcinoma

**DOI:** 10.1155/2014/153867

**Published:** 2014-04-28

**Authors:** Sheau-Fang Yang, Chien-Wei Chang, Ren-Jie Wei, Yow-Ling Shiue, Shen-Nien Wang, Yao-Tsung Yeh

**Affiliations:** ^1^Department of Pathology, Kaohsiung Municipal Ta-Tung Hospital, Kaohsiung City 801, Taiwan; ^2^Department of Pathology, Faculty of Medicine, College of Medicine, Kaohsiung Medical University, Kaohsiung City 807, Taiwan; ^3^Department of Medical Laboratory Sciences and Biotechnology, Fooyin University, Kaohsiung City 831, Taiwan; ^4^Cancer Center and Division of General & Gastroenterological Surgery, Department of Surgery, Kaohsiung Medical University Hospital, Kaohsiung City, Taiwan; ^5^Department of Pathology, Kaohsiung Armed Forces General Hospital, Kaohsiung City 802, Taiwan; ^6^Department of Biological Sciences, National Sun Yat-sen University, Kaohsiung City 804, Taiwan; ^7^Department of Surgery, Faculty of Medicine, College of Medicine, Kaohsiung Medical University, Kaohsiung City 807, Taiwan; ^8^Division of Hepato-Pancreatico-Biliary Surgery, Kaohsiung Medical University Hospital, Kaohsiung City 807, Taiwan

## Abstract

Hepatocellular carcinoma (HCC) has been known as one of the most lethal human malignancies, due to the difficulty of early detection, chemoresistance, and radioresistance, and is characterized by active angiogenesis and metastasis, which account for rapid recurrence and poor survival. Its development has been closely associated with multiple risk factors, including hepatitis B and C virus infection, alcohol consumption, obesity, and diet contamination. Genetic alterations and genomic instability, probably resulted from unrepaired DNA lesions, are increasingly recognized as a common feature of human HCC. Dysregulation of DNA damage repair and signaling to cell cycle checkpoints, known as the DNA damage response (DDR), is associated with a predisposition to cancer and affects responses to DNA-damaging anticancer therapy. It has been demonstrated that various HCC-associated risk factors are able to promote DNA damages, formation of DNA adducts, and chromosomal aberrations. Hence, alterations in the DDR pathways may accumulate these lesions to trigger hepatocarcinogenesis and also to facilitate advanced HCC progression. This review collects some of the most known information about the link between HCC-associated risk factors and DDR pathways in HCC. Hopefully, the review will remind the researchers and clinicians of further characterizing and validating the roles of these DDR pathways in HCC.

## 1. The Common Causes of Genetic Alterations and Genomic Instability in HCC

**Figure 1:**
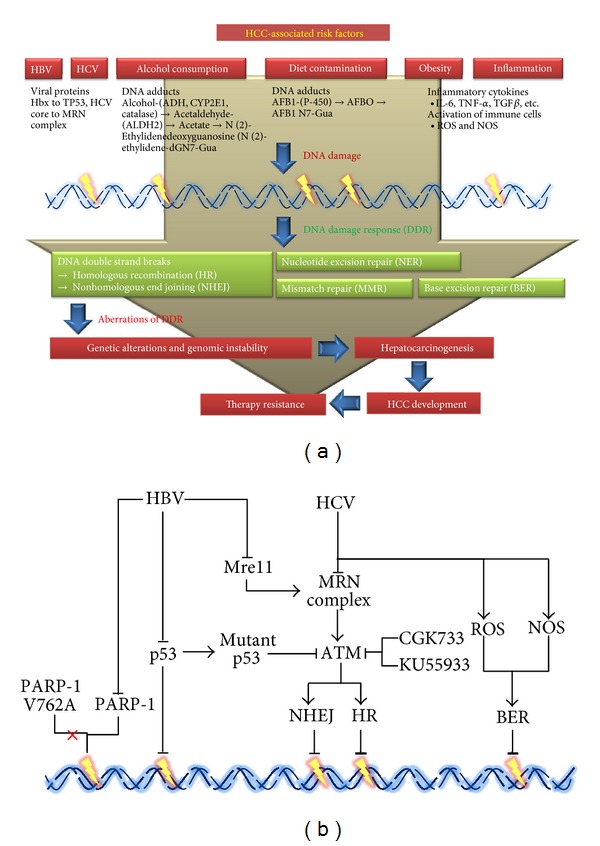
(a) A diagram of the potential involvement of DDR pathway in HCC. (b) Short summary of the link between HBV and HCV infections and DDR pathways in HCC.

Liver cancer is the sixth most common cancer overall but the third most frequent cause of cancer death [1, 2]. Among primary liver cancers, hepatocellular carcinoma (HCC), the major histological subtype, is associated with multiple risk factors, including hepatitis B and C virus (HBV and HCV) infection, alcohol consumption, obesity, and diet contamination (Figure 1). HCC frequently arises in the context of chronic injury and inflammation that promote DNA damage and chromosomal aberrations [3], which trigger a prompt set of signaling events known as the DNA damage response (DDR) pathways which coordinate DNA repair, cell cycle arrest, and ultimately cell death or senescence [4–6]. There are several types of DNA damage and corresponding repair mechanisms that have been implicated in HCC such as stalled DNA replication fork by homologous recombination (HR) [7], base mismatches by mismatch repair (MMR) [8], and the most serious form of DNA damage, double-strand break (DSB) [9], by nonhomologous end joining (NHEJ) [10, 11] (Figure 1). Aberrations of DDR may ravage genomic integrity, trigger hepatocarcinogenesis, and facilitate advanced HCC development. It is increasingly recognized that both genetic alteration and genomic instability are common features of human HCC. Hence, better understanding of the roles of DDR pathways may help us to develop the strategies for treatment or prevention of HCC. It will be worthy to remind people of the importance of DDR pathways in HCC.

Genomic instability including telomere erosion [12–14], chromosome segregation defects [15, 16], and alterations in the DDR pathways [17, 18] has been regarded as a common feature of human HCC. Various genetic alterations accumulate during hepatocarcinogenesis that change the signal transduction network [19]. Although different types of liver carcinogenesis models have been described [19–22], the direct link of genetic and epigenetic changes to this cancer remains undetermined. A failure to repair these genetic or genomic alterations probably resulted from DNA damages, caused by many risk factors, may lead to hepatocarcinogenesis and continuously contribute to further HCC progression. In addition to its direct impacts on genomic integrity, DNA damage can also increase viral DNA integration into the host genome [23]. Integration of HBV DNA and resulting induction of genomic instability is one of the proposed mechanisms suggested for HBV-related liver carcinogenesis [24]. In addition, HBV DNA integration can also occur in human satellite DNA sequences [25]; genomic repetitive sequences are suspected to be hotspots for HBV DNA integration [26].

Frequent mutations of coding nucleotide repeats are thought to contribute significantly to carcinogenesis associated with microsatellite instability (MSI), one kind of genomic instability. Microsatellite alterations have been widely and frequently reported, mainly when cirrhosis is associated with HBV infection [27–31]. However, the frequency of MSI in HCC tumors exhibits large variations. More than 10% (10–43%) of HCCs in European, American, Japanese, and black Southern African patients have been shown to exhibit MSI at one or more microsatellite markers [28–38], whereas this phenotype has rarely been observed in other European, American, Japanese, Taiwanese, Korean, and Chinese series of HCC tumors [39–47]. Several human cancers have been under extensive loss of heterozygosity (LOH), another kind of genomic instability, including breast cancer, ovarian cancer, renal cell carcinoma, HCC, lung cancer, and colorectal cancer; a number of deleted chromosomal regions that are important in those cancers have been identified [48–52].

Since with genetic alterations, genomic instability, and alterations of DDR pathways have been recognized as a common characteristic of HCC, we will shortly review their relationships with multiple risk factors, including hepatitis B and C virus infection, alcohol consumption, and diet contamination as follows.

### 1.1. Common Chemical Carcinogens in HCC Development

The aflatoxin-liver cancer risk relationship is one of the most documented examples of a disseminated environmental chemical carcinogen [53]. Aflatoxin B1 (AFB1), a well-established animal hepatocarcinogen, is a suspected HCC risk factor in hyperendemic areas like Sub-Saharan Africa, Southeast Asia, and Southern China, where dietary staples are highly contaminated [17]. Prospective epidemiological studies have shown a more than multiplicative interaction of HBV or HCV and aflatoxins in terms of HCC risk. Studies of the metabolism of AFB1 revealed that the compound is activated to its electrophilic DNA-binding form through an epoxidation pathway. Furthermore, activation and DNA binding produces identical DNA adduct profiles, with the N7 position of guanine representing the only site of adduct formation [18]. In regions where exposure to AFB1 and chronic exposure to HBV are frequently concomitant, there is a high incidence of HCC that often harbors* TP53* mutations [54]. It is possible that aflatoxin-induced DNA adducts are fixed as mutations due to the HBV-related increase in cell proliferation and hyperplasia, thus promoting the clonal expansion of mutant cells. Mutations of* TP53* such as the transversion in codon 249 (guanine (G) to thymine (T)), which causes an arginine (R) to serine (S) substitution, are present in 50% of HCCs [55, 56] and have been related to consumption of AFB1-contaminated food [57, 58]. Studies from Qidong and Guanxi, China, and South Africa revealed that the mutation occurs at the codon 249 hotspot in exon 7 of the* TP53* gene in HCC patients [57, 59–62]. The target organ for metabolism of AFB1 is the liver. Following ingestion with AFB1-contaminated food, AFB1 may be metabolized by cytochrome P450 enzymes to reactive genotoxic intermediates (aflatoxin B1-8, 9-oxide, AFBO) or hydroxylated (to AFQ1 and AFM1) and demethylated (to AFP1) to become less harmful than AFB1. The highly reactive genotoxic compound, AFBO, can bind to liver cell DNA as a result, and DNA adducts are formed, namely, 8,9-dihydro-8 (N7guanyl)-9-hydroxy-AFB1 (AFB1 N7-Gua) [63–66]. If this is not repaired before DNA replication, the DNA adducts interact with the guanine base of the DNA and cause mutational effects in the* TP53* tumor suppressor gene [63, 65, 67], resulting in hepatocarcinogenesis. Mutated R249S-TP53 protein expression may lead to inhibition of apoptosis, inhibition of TP53-mediated transcription, and stimulation of liver cell growth in vitro[55].

Malignant transformation of hepatocytes may occur as a consequence of various aetiologies, such as alcohol, in the context of increased cellular turnover induced by chronic liver injury, regeneration, and cirrhosis. Alcohol intake has been definitely recognized as a cause of chronic liver diseases, including HCC [68]. An indirect mechanism includes the development of cirrhosis, which is probably the most common pathway to liver carcinogenesis in developed countries [69]. Chronic alcohol use of greater than 80 g/day for more than 10 years increases the risk for HCC approximately 5-fold. Nevertheless, alcohol use of less than 80 g/day is associated with a nonsignificant increased risk for HCC [70]. The risk does not decrease with abstinence because HCC can also occur in a noncirrhotic liver. The mechanisms by which alcohol causes HCC are incompletely understood but may include chromosomal loss, oxidative stress, a decreased retinoic acid level in the liver, altered DNA methylation, and genetic susceptibility [68]. Acetaldehyde, the primary metabolite of ethanol, is suspected to play a major role in alcohol-related carcinogenesis. The binding of DNA and formation of DNA adducts represent one mechanism by which acetaldehyde could trigger the occurrence of replication errors or mutations in oncogenes or tumor suppressor genes [71, 72]. Thus, the development of acetaldehyde-derived DNA adducts may be an important mechanism in the carcinogenicity of alcoholic beverages [73]. Ingested ethanol is mainly oxidized by the enzymes alcohol dehydrogenase (ADH), cytochrome P-450 2E1 (CYP2E1), and catalase to form acetaldehyde, which is subsequently oxidized by aldehyde dehydrogenase 2 (ALDH2) to produce acetate. N (2)-Ethylidenedeoxyguanosine (N (2)-ethylidene-dG) is the major adduct formed in this reaction. Although some DNA adducts formation is demonstrated to be an initiation step of carcinogenesis [74, 75], it is still unclear whether these alcohol-related DNA adducts are true factors or initiators of cancer. Future studies are needed to better characterize and to validate the roles of these DNA adducts in human study.

### 1.2. The Link between Inflammation and ROS-Mediated DNA Damage to HCC Development

Activated inflammatory cells release free radicals, such as reactive oxygen species (ROS) and nitric oxide reactive species (NOS), which in turn can cause DNA damage and lead to gene mutations, thus fostering neoplastic transformation. Inflammation and oxidative stress associated with chronic active hepatitis may result in DNA damage and mutations. The chronic inflammation is characterized by the continued expression of cytokines and recruitment of immune cells to the liver. In fact, hepatic oxidative stress is also strongly associated with increased risk for HCC in patients with chronic HBV and HCV infections [76, 77]. On the other hand, obesity and inflammation-associated metabolic disorders are also able to promote increased secretion of cytokines (known as adipokines) by adipose tissue [78], as well as inflammatory cells, which include resident liver macrophages or Kupffer cells (KCs) [78, 79]. Hypertrophic adipocytes resulted from deposition and accumulation of excess lipids secrete free fatty acids (FFAs), and together with various immune cells which release various proinflammatory cytokines including tumor necrosis factor (TNF), interleukin-6 (IL-6), IL-1*β*, IL-8, IL-10, IL-18, and IL-17, as well as more specialized adipokines, such as leptin and adiponectin [78, 80–85]. Among all of these cytokines, IL-6 is both proinflammatory and a useful marker for obesity-associated inflammation. In the liver, IL-6 is mainly secreted by KC and hepatic stellate cells (HSC) and to a lesser extent by stimulated hepatocytes [86, 87]. Earlier studies have also revealed that both IL-6 and TNF increase hepatic production of C-reactive protein (CRP), a major acute phase protein, which is a nonspecific but sensitive marker of infection and tissue inflammation that is increased in obesity [88–90].

The balance of cytokines is also critical for regulating the type, extent, function, and outcome of inflammatory cell infiltrates. Profibrotic cytokines (IL-4 and IL-13) are produced by the increased population of NKT cells in chronically infected livers [91, 92]. Emerging data suggest that the inflammatory milieu represents a favorable niche for genetic mutations leading to cancer. A consequence of chronic inflammatory responses initiated by viral infection is the release of free radicals, such as ROS and NOS [93, 94]. The inflammatory response includes the activation of macrophage and neutrophil cells that result in a complex spectrum of chemically reactive species that damage DNA and other biomolecules [95]. Activated macrophages overproduce NO and superoxide (O_2*i*_^−^) that combine rapidly to form peroxynitrite (ONOO^−^). The latter decomposes to reactive intermediates that can cause damage to DNA and other biomolecules. The activated neutrophils, on the other hand, contribute to the myeloperoxidase-mediated generation of hypochlorous acid (HOCl)—a potent oxidizing and halogenating agent [95]. While many of the DNA lesions formed are oxidized forms of DNA bases themselves [96, 97], more bulky DNA lesions can also arise from the endogenous peroxidation of lipids that generate highly reactive aldehyde derivatives that react with DNA [98]. The generation of guanine radical intermediates also leads to the formation of cross-linking reactions with thymine [98, 99]. Intriguingly, NO could contribute to viral persistence by means of its antiapoptotic effect in hepatocytes and may itself induce viral mutations and promote a selective suppressive effect on Th1 cells [94]. NO can also directly influence liver cell survival by preventing apoptosis through activation of the nuclear factor kappa B (NF-*κ*B) signaling pathway [100]. ROS and other oxidative stress-related intermediates contribute to death, the perpetuation of chronic inflammatory responses, fibrogenesis, with a major focus on hepatic chronic wound healing, and liver fibrogenesis [101]. In addition, ROS are also involved in necrosis and apoptosis of hepatocytes and HSC activation [102, 103]. Together, these oxygen-derived free radical species are able to induce several alterations and structure/function modifications of cancer-related proteins and gene mutations, including those related to cell-cycle control, apoptosis, lipid peroxidation, and DNA repair.

The chronic inflammation affects many cellular pathways, leading to fibrosis and cirrhosis and finally hepatocarcinogenesis. Liver injury induces tissue repair and liver regeneration, which involve deregulated growth and death of hepatocytes. High cell turnover induces several critical alterations for malignant transformation, including structural and/or functional modifications of proteins involved in cell-cycle control, apoptosis, oxidative stress, lipid peroxidation, and DNA repair damage [104, 105]. Moreover, TNF-*α*-induced NF-*κ*B activation plays a key role in hepatocarcinogenesis [106, 107].

Several major classes of free radical scavengers, such as catalase, superoxide SOD, and glutathione peroxidase (GSH-P), were investigated in various types of liver damage, and they afforded effective protection against the oxidative insults to hepatic parenchyma [108]. High levels of ROS, from phagocytic cells, such as KCs, protect the organism from external pathogens; however, lower amounts of ROS mainly from HSC actively participate in the regulation of intracellular signaling [103, 109]. Platelet-derived growth factor (PDGF) is the most potent mitogen of HSC and is, therefore, likely to be an important mediator during liver fibrogenesis [110]. Interestingly, NAD(P)H is expressed in HSC and produces ROS, which, in turn, induces the production of PDGF; again, this molecule increases mitosis of HSC [109]. These results strongly suggest that ROS plays an important role in fibrogenesis increasing PDGF throughout.

## 2. The Impact of DDR Pathways on HCC Development

It has been revealed that aberrations of DNA repair proteins such as hOGG1, XRCC1, TP53, Poly(ADP-ribose) polymerase 1 (PARP-1), Mre11-Rad50-Nbs1 (MRN) complex, and ataxia telangiectasia-mutated (ATM) kinase were correlated with HCC [111–117]. Lack of Ku70, a DNA repair protein that takes part in NHEJ pathway, showed high proliferative activity with increased cyclin D1 and loss of TP53 via proteasomal degradation, thus suggesting that defects in NHEJ pathway lead to chromosomal instability and facilitate development of HCC [118]. In addition, MRN complex, a DSB sensor [119] and DNA binding protein [120], recruits ataxia ATM to participate in two major DNA repair pathways that are responsible for DSB: HR and NHEJ [121–123]. It has also been reported that mutant TP53 can interact with Mre11 and thereby impair the proper function of MRN complex and subsequent activation of ATM [124]. Moreover, inactivation of DNA repair contributes to development of HCC has been revealed [125, 126].

Base excision repair (BER) pathway constitutes the primary defense mechanism against lesions generated by DNA damaging agents like viruses [127]. Two DNA repair genes, hOGG1 and XRCC1 that encode DNA glycosylase enzyme [113] and scaffold protein [128] responsible for BER pathway, have been implicated with hepatitis virus-related liver cancer [129–131]. Furthermore, genetic polymorphisms of DNA repair genes reduce DNA repair capacity [132]. For instance, hOGG1, Cys326Cys, and Ser326Cys genotypes' XRCC1 codon 399 variant Arg/Gln allele has been recently shown to be associated with increased HCC risk (Table 1) [111, 112]. Recent study has also reported that Ser326Cys polymorphism in hOGG1 gene is associated with HCC carcinogenesis in Eastern Asians [113]. The nucleotide excision repair (NER) process is the most important repair pathway for elimination of DNA damage caused by exogenous agents including UV light, DNA-adducing carcinogens, and some endogenously generated oxidative lesions [133]. NER is a multistep process involving more than 25 polypeptides; it consists in an initial damage recognition step, followed by an incision to either side of the lesion and the removal of the damage-containing oligonucleotide, DNA repair synthesis, and finally reseal of the strand by a DNA ligase [134]. Overexpression of two key genes involved in the early steps of the NER process, ERCC1 and XPC, is associated with liver fibrogenesis and cancer and could be related to the well-recognized resistance of HCC to chemotherapeutics [135]. Several studies have revealed multiple genetic alterations of the mismatch repair (MMR) genes in hepatocarcinogenesis [136]. Among the MMR genes, human Mut L homologues 1 and 2 (hMLH1 and hMSH2) play important roles in DNA repair [137–139]. Immunohistochemical studies of MMR genes in HCC are few [40, 140–143]. HCC lacking both O6-methylguanine-DNA methyltransferase (MGMT) and hMLH1 is correlated with an advanced stage and a poor prognosis.* Mgmt*-targeted (*Mgmt* ^−/−^) mice treated with alkylating agents develop HCC [144]. Reduced expression of hMSH2 and hMLH1 in both HCC and ANCLT suggests that this event occurs at early stages of HCV-related hepatocarcinogenesis. Moreover, the significant association between reduced expression of both MMR genes and poor histologic grades of the tumor claims that these proteins are involved in the process of cancer progression [136].

Although MRN complex has crucial physiologic activities in cell response to DNA damage, the role of defective MRN complex in HCC is still rarely described. A current study has reported that HBV infection downregulates the protein level of Mre11, and HCV core protein can interfere the formation of MRN complex by binding on Nbs1 and inhibit subsequent activation of ATM and other DNA repair proteins [116, 117]. Nevertheless, patients with occult HCV infection have been suggested to have high phosphorylated ATM, ATR, H2AX, and TP53, along with active recruitment of MRN complex, and further result in Bax-triggered mitochondria-mediated cellular apoptosis in lymphocyte [145]. These studies raised a question: which is a better strategy, to activate or to inhibit ATM activity, in treating HCC patients? Recently, Wang et al. revealed that GL331, a topoisomerase II inhibitor, activates autophosphorylation of ATM at S1981 and extends activations of DNA damage signaling pathways including TP53/p21 and Chk2/Cdc25A cascades to reach S phase arrest in HepG2 cells (Table 1) [146]. A small molecular inhibitor, CGK733, which targets kinase activity of ATM, can obviously reverse the resistance of HepG2 to taxol treatment (Tables 1 and 3) [147]. Besides, a synthetic cytotoxic effect was obtained in HepG2 cells when cotreated with sorafenib (multikinase inhibitor) and KU55933 (ATM inhibitor) (Tables 2 and 3) [148]. These results suggest that the ATM signaling pathway may serve as a novel target for the clinical treatment of HCC patients with chronic hepatitis B.

Genomic instability has long been proposed to be a mechanism by which a cell may acquire the necessary properties for invasion and metastasis [149]. The tumor suppressor TP53 has been well known in maintaining a stable genome under the toxic insults through its role in promoting cell-cycle checkpoints, DNA repair, and apoptosis [149]. It has been revealed that mutated* T53* gene was found in approximately 50% of hepatoma cells [114]. And mutant TP53 often gains additional oncogenic functions that endow cells with growth and survival advantages and differences in radiosensitivity [150, 151]. Recently, investigators have revealed that TP53-mutated cells provide much more resistance to radiotherapy than TP53 null (Hep3B) and wild type TP53 cells (HepG2), suggesting the loss or mutation of TP53 proteins produced radioresistance [115]. It has been also reported that mutant TP53 can interact with Mre11 and thereby impair the proper function of MRN complex (Table 1) [124]. Abrogation of Mre11 function limits the phosphorylation and activation of ATM, resulting in bypassing of the G2/M DNA damage checkpoint. Loss of this checkpoint severely reduces the ability for efficient HR, resulting in less conservation of genetic information and accumulation of DNA damage and genetic errors [124].

PARP-1 is encoded by ADP-ribosyltransferase (*ADPRT*) gene, which regulates gene transcription, cell death signaling, and DNA repair through production of the posttranslational modification poly(ADP-ribose) [152], and its activation is one of the early responses to DNA damage [153]. Aberrant protein residue at V762A in PARP-1 has defective cellular response for oxidative DNA damage and contributes to prostate cancer susceptibility (Table 1) [154]. In addition, a recent study has showed that alteration of PARP-1 at residue A762A significantly depresses PARP-1 activity and is related to increased risk of cervical cancer (Table 1) [155]. In HCC, HBV may confer the development of HCC via octameric tandem repeats that can bind on PARP-1, and this interaction suppressed the DNA repair activity of PARP-1 and even increased the replication efficiency of HBV [156].

## 3. Interplay between Viral Infection/Protein and DDR Pathways in HCC

The relation of the virus to the development of HCC is through chronic hepatitis and cirrhosis [157]. A study directly assessing 8-oxo-deoxyguanosine in human liver revealed that both HBV and HCV infections were associated with DNA damage [158]. Almost all patients with virus related HCC have an underlying cirrhosis. The hepatocyte necrosis and mitosis of chronic hepatitis favor nodular regeneration which, in appropriate circumstances, is followed by hepatocyte dysplasia and carcinoma [159]. Although nodular regeneration and cirrhosis remain the most important antecedents, the tumor can develop in the absence of cirrhosis. In this case, and by analogy with the HBV-like woodchuck chronic hepatitis, necroinflammatory activity may be an important requisite [160].

### 3.1. HBV

Chronic infection with HBV is a major risk factor for the development of HCC. The virus encodes a 17 kDa protein, HBx, which is thought to be involved in the development of HBV-associated HCC [161]. Epidemiologic studies have shown that chronic HBV infection is associated with a 100-fold increase in the risk for HCC development relative to noncarriers, placing HBV in the forefront among known human carcinogens [162, 163]. The integration of HBV DNA into hepatocytes is an integral step for persistent viral infection that leads to chronic hepatitis B infection, which ultimately causes HCC [164]. Viral DNA integration rearranges both host and viral genes, leading to the production of altered protein products and making hepatocytes more susceptible to HCC [165]. In addition, the insertion of viral genome may result in chromosome deletions and other general genomic instability [24] that activates several pathways switching on HCC development [166]. As HBV is a DNA virus which integrates its genome inside the host genome, during HBV infection, viral replication occurs inside infected hepatocytes within viral capsids. In this manner, viral genome conceals itself from pattern recognition receptors (PRRs), of innate immune system, preventing the detection of initial HBV infectious particles [167] PPRs including Toll-like receptors (TLRs) [168, 169] that recognize the pathogen-associated molecular patterns leading to a change of macrophage phenotype. These macrophages secrete ROS, such as type I interferons (IFNs) (IFN-*α* and IFN-*β*), NO, and other cytokines and chemokines. It was studied that the general production of NO and ROS by activated macrophages may also cause hepatocytes destruction [170]. ROS can cause oxidative protein and DNA damage [171]. ROS also affects the central cellular processes such as apoptosis and proliferation leading to the development of cancer [172]. Genes that are involved in DNA damage such as CCND1, CDKN1A (p21CIP1/WAF1), MSH2, MSH3, TP53, and XIAP [173] along with modified gene expression and mutations are all required participants in the process of carcinogenesis. It was found that oxidative stress is associated with hepatitis B activity and XRCC1 gene is putatively associated with DNA damage [112].

Studies with transgenic mice provide conflicting results. Some X protein of HBV (HBx) transgenic mice develop liver cancer [174], while others do not [175–177]. However, HBx can serve as a cofactor for HCC in those transgenic mice that do not develop spontaneous tumors [176, 178, 179]. A cofactor role for the X protein is also observed in woodchuck hepatitis virus transgenic mice that do not spontaneously develop liver tumors [180]. Integrated viral DNA sequences might act in cis to modify host gene expression or encode viral proteins that may interfere with normal cellular functions either directly or indirectly. Accordingly, the inactivation of cellular proteins by viral proteins has been proposed as a potential mechanism of malignant transformation of hepatocytes by HBV [181]. Recent studies have shown that the HBx binds to cellular TP53 [182, 183] and, in vitro, inhibits TP53 sequence-specific DNA binding, TP53 transcriptional activity, and its association with the transcriptional factor ERCC3. Previous discussion showed that HBx antigen also participates in processes that are associated with the progression of chronic liver disease, including angiogenesis and fibrosis. Moreover, HBx expression affects DNA repair in a TP53-dependent manner [184]. Binding of HBx on TP53 leads to inhibition of TP53-dependent DNA repair, thereby DNA damage accumulation in HBV-infected cells, and subsequently depressed TP53-dependent apoptosis [183, 185, 186]. Furthermore, recent study has also indicated that HBsAg disrupts promyelocytic leukemia- (PML-) mediated DSB HR repair or apoptosis, which may facilitate hepatocarcinogenesis [18]. Based on these data, it has been suggested that HBV may affect a wide range of TP53 functions [183]. ATM and ATR kinases phosphorylate Ser-15 and Ser-20 of p53 following DNA damage [187, 188]. HBx also sensitizes hepatocytes to p53 apoptosis via activation of the p38MAPK pathway, which couples p53 stabilization and p53 activation, by E2F1 induction and ATR activation, respectively [189]. In addition, HBx can impair the ATR-dependent phosphorylation of Chk1 and monoubiquitination of FANCD2. Overexpression of ATR reverts the MMC-induced phenotype of Chk1 and FANCD2 in HBx transformed cells [190]. The defect of intra-S-phase checkpoint may further result in accumulation of genomic instability. Many pathways of cellular immune system are activated during HBV infection. The deregulation of signaling pathways including MAPKs, TP53, sex steroid, Wnt/*β*-catenin, transforming growth factor *β* (TGF*β*), PI3 K/AKT, cytokines, NF-*κ*B, and Hedgehog (Hh) were found to be closely related with HCC development [191]. In addition, HBV heat shock proteins can upregulate three dominant groups of genes including oxidative and metabolic stress and growth and apoptosis-related genes [192]. The level of inflammatory cytokines such as IL-6 is also found to be increased in HCC cells [193]. Level of IL-6 may also predict the shift from viral hepatitis to HCC in humans [194] due to Hh signal activation. It has been documented that the expression of HBx and Hh is highly correlated in human liver cancer cell lines [195].

### 3.2. HCV

HCV infects more than 170 million people in the world. The importance of HCV infection in HCC and non-Hodgkin's B-cell lymphomas has been well documented [196, 197], indicating that HCV is a unique nonretroviral oncogenic RNA virus. From the genetic perspective, cancer represents the phenotypic consequence of the accumulation of mutations or deletions of DNA involving the inactivation of tumor suppressor genes and the activation of oncogenes by a stepwise process. It has recently demonstrated that HCV infection induces DNA damage that leads to a mutator phenotype [198]. In addition, HCV infection is able to cause DSBs and to enhance the mutation frequency of cellular genes, including protooncogenes and immunoglobulin genes [199]. HCV-induced inflammation and oxidative stress have been also implicated as risk factors for liver damage and cancer development [200–202]. Patients with greater intrahepatic oxidative stress have a higher incidence of HCC recurrence [203]. Generally, HCV infection can induce the production of total NO, that is, NO_*X*_ which includes both nitrites (NO_2_^−^) and nitrates (NO_3_^−^); there is an association between the viral load and the level of NO_*X*_ in the serum of HCV-infected patients [204]. Enhanced levels of inducible nitric oxide synthase (iNOS) expression (implying excessive NO formation) in the liver of HCV-infected patients also positively correlated with viral load and severity of hepatic inflammation [94]. In several human gastrointestinal neoplasms, including HCV-associated HCC, the immunologic (type II) isoform of NO synthase (NOS), that is, inducible NOS (iNOS), which generates NO from L-arginine in inflamed tissues, is elevated [205]. The enhanced levels of iNOS in HCV-associated T lymphocytes correlated with the high level of expression of HCV proteins [206]. Other observations supported this finding [207–209] and suggested that an important treatment option may include modulation of NO metabolism to prevent HCC pathogenesis and improve patient outcomes [207]. Therefore, one of the means by which HCV exerts its effects upon infected cells is likely oxidative stress, including NO production. Furthermore, Maki et al. [203] demonstrated that HCV-induced inflammation causes oxidative DNA damage and promotes hepatocarcinogenesis. In particular, generation of ROS and NOS associated with the decreased antioxidant defense seemed to promote the development and progression of hepatic and extrahepatic complications of HCV infection [210].

The HCV genome contains a large open reading frame encoding a polyprotein precursor of 3,010 to 3,033 amino acids and an untranslated region at the 59 and 39 ends of the genome. This polyprotein is processed into at least 10 proteins, 4 of which are structural proteins (core, E1, E2, and p7) and 6 nonstructural (NS) proteins (NS2, NS3, NS4A, NS4B, NS5A, and NS5B) in the order from the 59 end [211–214]. HCV core protein activated SRE-, NF-*κ*B-, and AP-1-associated signals, and NS4B protein activated the NF-*κ*B-associated signal [100]. Interestingly, HCV core protein also binds to the Nbs1 protein and inhibits the formation of the Mre11/Nbs1/Rad50 complex, thereby affecting ATM activation and inhibiting DNA binding of repair enzymes. These data indicate that HCV infection inhibits multiple DNA repair processes to potentiate chromosome instability in both monocytes and hepatocytes. These effects may explain the oncogenicity and immunological perturbation of HCV infection [117]. Moreover, replication of both genome-length HCV RNA (HCV-O, genotype 1b) and the subgenomic replicon RNA is notably suppressed in ATM- or Chk2-knockdown cells. HCV NS3-NS4A interacted with ATM and HCV NS5B interacted with both ATM and Chk2. These results suggest that the ATM signaling pathway is critical for HCV RNA replication and may represent a novel target for the clinical treatment of patients with chronic hepatitis C [215].

## 4. DDR-Based Therapy Resistance in HCC

Dysfunction of one DNA repair pathway may be compensated for by the function of another compensatory DDR pathway, which may be increased and may contribute to resistance to DNA-damaging chemotherapy and radiotherapy [10]. Human cancers exhibit genomic instability and an increased mutation rate due to underlying defects in DNA repair. Cancer cells are often defective in one of six major DNA repair pathways, namely, mismatch repair (MMR), BER, nucleotide excision repair (NER), HR, NHEJ, and translesion synthesis (TLS) [216]. Dysregulation of DNA damage repair and signaling to cell-cycle checkpoints, known as the DDR, is associated with a predisposition to cancer and affects responses to DNA-damaging anticancer therapy.

HCC has been known as one of the most lethal human malignancies, due to the difficulty of early detection, chemoresistance, and radioresistance [217, 218], and is characterized by active angiogenesis and metastasis, which account for rapid recurrence and poor survival [219]. Transarterial embolization (with or without chemotherapy) has been a general treatment choice for unresectable HCC through and is the standard of care for patients with preserved liver function and asymptomatic, noninvasive multinodular HCC confined to the liver [220, 221]. However, hypoxia also has been revealed to be associated with proliferation, angiogenesis, epithelial mesenchymal transition (EMT), a critical process for tumor invasion, and metastasis as well as chemoresistance and radioresistance in HCC [219, 222, 223]. Additionally, HCC is unique as it frequently reoccurs after treatment irrespective of the different etiological factors including HBV and C HCV, alcohol abuse, and nonalcoholic steatohepatitis [224–226]. One possible reason for the frequent recurrence of HCC might be due to many patients being affected with hepatitis virus-associated chronic liver inflammation [224, 227].

DNA damaging agents are widely used in treatment of hematological malignancies and solid tumors [228]. However, DDR also protects against genomic instability, and may enable cancer to become resistant to IR and chemotherapy drugs by enhanced DNA repair of the lesions [229, 230]. In addition, the development and progression of HCC is significantly correlated to the accumulation of genomic alterations [231]. Sonic hedgehog (Shh) ligand induced signaling is critical to embryogenesis and resistance to chemotherapy. Recent study showed that HCC cells, HA22T, and Sk-Hep1 are resistant to radiotherapy upon treatment of Shh ligand and reduced the radiotherapy-induced phosphorylation of Chk-1 as well as impairing the repair of DNA DSB. This phenomenon can be partly reversed by Shh antibody neutralization, suggesting that activation of Shh signaling protects HCC cells against ionizing radiation in an autocrine manner. Impairment of DNA damage repair might involve mechanism of Shh-induced radioresistance [232]. Inhibition of G2 DNA damage checkpoint by UCN-01 also promotes the cytotoxicity of paclitaxel in paclitaxel resistant HCC cells (SNU449, SNU398, SUN368, SNU354, and HepG2 cells) [233]. Excision repair cross complementation group 1 (ECCR1) belong to the nucleotide excision repair (NER) pathway and are essential to the repair of cisplatin DNA adducts [234]. Ueda et al. indicated that high nuclear staining of ECCR1 was found in 33% of paraffin-embedded HCC tissue (*n* = 43), which is positively associated with succinic dehydrogenase, a major target aimed by cisplatin in mitochondria [235] and resistance to treatment of cisplatin [236]. A previous study has shown that overexpression of two NER genes, ERCC1 and XPC, is associated with liver fibrogenesis and cancer and could be related to the well-recognized resistance of HCC to chemotherapeutics [135]. It has been implicated that molecules involved in DNA repair or cell cycle checkpoints can be targets to enhance tumor radiosensitivity [237]. Taken together, these observations suggest that the drugs targeting DNA repair related proteins may be applicable to improve radioresistance and chemoresistance of HCC.

## 5. Conclusions

The accumulation of genetic alterations and genomic instability, partly resulted from causing DNA damages of HCC-associated risk factors and a concomitant failure of repairing lesions, is currently regarded as a common feature of HCC [231]. These risk factors have been proposed directly or indirectly to form DNA adducts and to produce ROS and NOS, which in turn cause DNA damage and lead to gene mutations, thus fostering neoplastic transformation. In addition, they can also target certain genes of the DDR pathways through indirect induction of multiple oncogenic signaling to counter the DDR pathways or through their own proteins to interrupt the DDR genes. For instance, it is now clear that HBV, probably through HBx, potentiates damage to DNA in infected cells, probably by inhibiting various aspects of the DDR. As many other viruses, HBV inactivates TP53 following infection. HBx binds directly to TP53 and inhibits its transcriptional activation properties [182, 238]. It also inhibits the association of TP53 with transcription factors such as ERCC3/XPD and ERCC2/XPB [183, 239] and, as a consequence, abrogates TP53-induced apoptosis [183]. Since TP53 plays a key role in maintenance of the integrity of the genome, such a mechanism of TP53 inactivation would establish a direct link between HBV infection and the malignant transformation of hepatocytes. More interestingly, DNA damages can also increase viral DNA integration into the host genome [23] and further induce genomic instability [24–26]. These studies straightly point out the importance of these risk-factors-associated genetic alterations, genomic instability, and alterations of the DDR pathways in HCC development and treatment. On the other hand, the reason why HCC is one of the most lethal human malignancies is at least in part due to its easy production of chemoresistance and radioresistance [217, 218]. Although DDR may protect against genomic instability, DDR also helps cancer to become resistant to IR and chemotherapy drugs by enhancing DNA repair of the DNA lesions [229, 230]. Furthermore, various DDR pathways can compensate for each other and contribute to resistance to DNA-damaging chemotherapy and radiotherapy [10, 229, 230]. It has been implicated that molecules involved in DNA repair or cell cycle checkpoints can be targets to enhance tumor radiosensitivity [237].

Taken together, a better understanding of the involvement of DDR in HCC may help us to develop the strategies for treatment or prevention of HCC. The development of drugs targeting DDR pathways may be applicable to improve radioresistance and chemoresistance of HCC and further prolong their survival. Herein, we present the critical involvement of the DDR pathways in HCC. Hopefully, this review will enforce the researchers and clinicians to further explore and validate the impacts and underlying mechanisms of DDR pathways in HCC.

## Figures and Tables

**Table 1:** Summary of aberrations of the DDR pathways and their subsequent effects.

DNA repair protein	Mutation or polymorphism	Effect	Reference
OGG1	Ser 326 Cys	Increased risk of HCC.	[112]

XRCC1	Arg 280 His	Increased susceptibility to HBV infection.	[112]
	Arg 399 Gln	Increased risk of HCC.	[111]
	Arg 194 Trp and Arg 280 His	Increased risk of bladder cancer.	[240]

TP53	Arg 273 His, Arg 175 His, and Cys 135 Tyr	TP53 GOF mutants stimulate EMT features through binding to and transrepressing the promoter of *miR-130b* in endometrial cancer cells.	[241]
	Arg 248 Trp	TP53 GOF mutant interacts with the nuclease Mre11 and suppresses the loading of the MRN complex to DNA DSB, subsequently impairing the activation of ATM.	[124]
	Alteration of protein residues in a.a. 302–320	Retained associate with topoisomerase I and induced its activity during times of DNA stress in a regulated fashion, facilitating DNA repair. Suggested to lead to inappropriate topoisomerase I activity, resulting in an increase in recombinogenic events.	[242]

PARP-1	Val 762 Ala	Depressed PARP-1 activity is related to increased risk of cervical cancer, smoking-related lung cancer, and prostate cancer susceptibility.	[154, 155, 243]

Mre11	C 1714 T	Mutant *Mre11* found at codon 1714C→T, which encodes a prematurely truncated protein, is suggested to be eliminated by NMD.	[244]

Rad50	Ser 635 Gly	Rad50 phosphosite-specific mutant supported normal activation of ATM in Rad50-deficient cells but failed to correct radiosensitivity, DNA DSB repair, and an S-phase checkpoint defect in Rad50-deficient cells.	[245]
	Hook domain replace with six a.a. residues from 684 to 689: Asn-Ala-Ala-Ile-Arg-Ser	Rad50 zinc hook mutant leads to MRN complex which failed to load to chromosomal DSB and exhibits very limited recruitment of DNA repair proteins.	[246]

Nbs1	657del5	The *Nbs1 657del5* founder allele is associated with an increased risk of breast cancer.	[247]
	Ser 706 X	Results in a premature stop at codon 706 and a truncated Nbs1 protein that lacks the extreme C-terminal ATM recruitment motif (ARM).	[248]

ATM	Ser 367 Ala and Ser 2996 Ala	Both the S367A and S2996A mutants were defective in correcting radioresistant DNA synthesis in A-T cells.	[249]
	Ser 1893 Ala	Defective activation of ATM was manifested as defective substrate phosphorylation of TP53, Chk2, Nbs1, and SMCI in A-T cells transfected with ATM S1893A mutant failed to correct radiosensitivity, radiation-induced chromosome aberrations, and the defective G2/M checkpoint.	[250]
	Ser 1981 Ala	ATM is sequestered with a dimer or multimer with its kinase domain bound to an internal domain of a neighbouring ATM molecule containing serine 1981. A mutation occurs at autophosphorylation site, which leads to ATM unable to be released from other ATM molecules, and fails to phosphorylate other cellular substrates while DNA is damaged.	[251]

**Table 2:** Roles of DNA DSB repair proteins in HCC.

DSB repair related proteins	Functions	Reference
p53 binding protein 1 (53BP1)	53BP1 enhances TP53-mediated transcriptional activation via binding on DBD of TP53. 53BP1 is also used as an indicator of DNA damage and has been shown to rapidly localize to regions of DNA double-strand breaks. 53BP1 has a binding site for phosphorylated H2AX and colocalizes with phosphorylated H2AX at sites of damage.	[252, 253]

Phospho-H2AX (*γ*H2AX)	H2AX is a potential regulator of DNA repair and is a useful tool for detecting DNA damage, which also frequently occurs in preneoplastic lesions of HCC.	[186, 254]

Ku70 (XRCC6)	Toll-like receptor 4 (TLR4) protects against HCC carcinogenesis by enhancing the expression and function of DNA repair protein Ku70. Ectopic expression of Ku70 protects against HCC initiation and progression by restoring the cellular senescent response, decreases DNA damage, and promotes programmed cell death in TLR4-deficient livers.	[255, 256]

Ku80 (XRCC5)	Overexpression of Ku80 obviously inhibits cell proliferation ability of HCC cells, SMMC7721, in vitro and in vivo, through functions as a tumor suppressor by inducing S-phase arrest in a TP53-dependent pathway.	[257]

DNA-PKcs	Tissue staining results showed that the highest ratio of DNA-PKcs positive expressing cells was detected in HCC than in cholangioadeno carcinomas biliary cystadenocarcinomas.	[258]

Ataxia telangiectasia mutated kinase (ATM)	Autophosphorylation of ATM at S1981 extends activations of DNA damage signaling pathways to reach S phase arrest in HepG2 cells. Inhibited activity of ATM improves the cytotoxicity of taxol and serafenib in HepG2 cells.	[146–148]

**Table 3:** Summary of the involvement of the DDR pathways in potential therapy resistance in HCC.

Protein	DNA repair pathway	Underlying effects	Reference
Chk-1	HR	Shh ligand abolished RT-induced phosphorylation of Chk-1 as well as impairing the repair of DNA DSB, which results in HCC cells, HA22T, and Sk-Hep1, resistant to radiotherapy.	[232]

Chk-2		UCN-01 promotes the cytotoxicity of paclitaxel in paclitaxel resistant HCC cells (SNU449, SNU398, SUN368, SNU354, and HepG2 cells) via inhibiting activity of Chk-2.	[233, 259]

ECCR1	NER	HCC patients with increased nuclear staining of ERCC1 are positively correlated with resistance of cisplatin treatment.	[236]

XPC		Increased XPC is associated with liver fibrogenesis and cancer and could be related to the well-recognized resistance of HCC to chemotherapeutics.	[135]

ATM	HR or NHEJ	Small molecular inhibitor, CGK733, which targets kinase activity of ATM can obviously reverse HBV-positive HCC cells,	[147]
		KU55933, ATM inhibitor cotreat with sorafenib, multikinase inhibitor, shows synthetic cytotoxicity to HepG2 cells.	[148]
